# Supplementary material for: Impact of nutrient warning labels on Colombian consumers’ selection and identification of food and drinks high in sugar, sodium, and saturated fat: A randomized controlled trial
Source: PLoS One. 2024 Jun 10;19(6):e0303514. doi: 10.1371/journal.pone.0303514 (PMC11164358; doi:10.1371/journal.pone.0303514)
Supplement: S1 Table — (DOCX) [file pone.0303514.s002.docx]

| **Stimuli Development** | | |
| --- | --- | --- |
| **Check** | **Instructions** | **Initial when complete** |
| Determine nutrition facts for mock products | 1. Two Registered Dietitians (RDs) separately determine the nutrition facts for mock products based upon a pre-determined, existing product. 2. RDs meet to review and compare nutrition facts for the mock products, resolving any discrepancies. 3. RDs finalize nutrition facts. |  |
| Calculate labels for mock products | 1. Two RDs separately review labeling protocols and calculate and assign labels for mock products. 2. RDs meet to review and compare Nutri-Score, Guideline Daily Amounts (GDA), and Nutrient Warning calculations and assignments, resolving any discrepancies. 3. RDs finalize labels. |  |
| Approval of nutritional information and labels | 1. Study team reviews final nutritional information and assigned labels and issues approval. |  |
| Create labels | 1. Graphic designer designs labels and applies to mock products. |  |
| Review mock products for correct criteria | 1. One study team member compiles all product images with labels applied. 2. Study team member writes out all information listed below for each product in each labeling condition. Study team member compares this information to the final nutrition facts and labeling spreadsheet, indicating “Yes” or “No” if information matches, noting any errors.    1. Package Size    2. Placement of Label    3. Color/Shape of Label    4. Label Text |  |
| Approval of final stimuli | 1. Study team reviews final product images and issues approval. |  |

| **Survey** | | |
| --- | --- | --- |
| **Check** | **Instructions/Notes** | **Initial when complete** |
| Confirm all study outcomes and demographics are being collected | 1. Make a list of all outcome variables and demographics being collected according to the final codebook. 2. Make a list of all outcome variables and demographic variables being collected in the survey. 3. Compare lists to ensure no variables are missing. |  |
| Pre-test survey | 1. Distribute survey to 5-6 team members, including at least 1 team member not involved in the project. 2. Ask team members to complete the survey once as if they were a study participant and note any issues (e.g., spelling, grammar, image resolution). 3. Ask team members to complete the survey an additional 1-2 times while comparing the survey to the codebook and note any issues (e.g., items not displayed as detailed in codebook). |  |
| Survey read-through | 1. Two study members assigned to complete the survey readthrough. One team member completes the programmed survey in Qualtrics, reading all text aloud. Other team member reads along in final codebook, ensuring that all text and survey programming match. Repeat for each trial arm. 2. Read stimuli text aloud to check for errors. 3. Compare all stimuli in survey to final images to ensure the programmed images match the study design. |  |
| Review survey flow | 1. Ensure randomization occurs at the start of the experiment. 2. Ensure study blocks are displayed in the correct order. |  |
| Generate test data | 1. Upload test data into Stata. 2. Ensure randomization is working correctly. 3. Ensure question labels and response coding match the codebook. |  |
| Final survey set-up review | 1. Confirm that the correct quotas are set up. 2. Remove IP address collection. 3. Update redirect URLs with survey research panel information. |  |
