# Supplementary material for: Impact of nutrient warning labels on Colombian consumers’ selection and identification of food and drinks high in sugar, sodium, and saturated fat: A randomized controlled trial
Source: PLoS One. 2024 Jun 10;19(6):e0303514. doi: 10.1371/journal.pone.0303514 (PMC11164358; doi:10.1371/journal.pone.0303514)

| **Construct (variable name) [reference]** | **Item** | **Response scale** |
| --- | --- | --- |
| **Screener** | | |
|  | Welcome! Thank you for your interest in our research study. Please answer the following questions so we can find out if you are eligible to participate.  Do you agree to take the screening survey? Click the arrow to continue if you agree.  Le damos la bienvenida. Gracias por su interés en participar en nuestro estudio. Responda a las siguientes preguntas para que podamos determinar si cumple los requisitos necesarios para participar.  ¿Quiere hacer la encuesta de elegibilidad? Pulse la flecha si quiere continuar.  [page break] |  |
| Age (age) | What is your age (in years)?  ¿Cuál es su edad (años cumplidos)? | [text entry]  *[if younger than 18 or older than 64, ineligible]*  **require response* |
| Gender (gender) | How would you describe your gender identity?  ¿Cómo describe usted su identidad de género? | 1=Masculine  2=Feminine  3=Other, please specify: _____  1 =Masculino  2= Femenino  3 = Otro, por favor especifique: _____  *[quota: 49% male, 51% female]*  **require response* |
| Education (education) [1, 2] | What is the highest education level that you have completed?  ¿Cuál es el nivel educativo MÁS alto que usted ha alcanzado?  [page break] | 1 = None  2 = Primary school  3 = 9^th^ grade  4 = High school diploma  5 = Associate’s degree  6 = Bachelor’s degree  7 = Graduate degree  1 = Ninguno  2 = Básica primaria (5^0^ grado)  3 = Básica media (9^0^ grado)  4 = Título de bachiller (11^0^ grado)  5 = Superior técnica  6 = Superior universitaria  7 = Posgrado  *[quota:*  *50% answer 1-4*  *50% answer 5-7]*  **require response* |
| Eligibility message | **Thank you for answering the questions. You are eligible to participate. Please, click the arrow to read the consent.**  **Gracias por contestar las preguntas. Usted es eligible para participar. Por favor, pulse la flecha para leer el consentimiento.** |  |
| **Initial prompts and questions** | | |
| Consent | [consent form]  *[page break]* |  |
| Instructions | **Please read each question carefully. You will not be able to change your answers after you advance to the next page.**  **This survey seeks your opinion. There are no right or wrong answers.**  **Por favor lea cada pregunta cuidadosamente.**  **Usted no podrá cambiar sus respuestas después de avanzar a la siguiente página. En adelante, seleccione una respuesta.**  **Esta encuesta busca conocer su opinión, no hay respuestas correctas ni incorrectas.**  *[page break]* |  |
| Randomization | *[Randomize participants to one of 4 arms]*  *Arm 1 (Control)*  *Arm 2 (Nutriscore)*  *Arm 3 (Octagon)*  *Arm 4 (GDA)* |  |
| **Fruit Drinks Selection Task** | | |
| Prompt | **The next questions are about drink products.**  **Las siguientes preguntas son sobre bebidas.**  *[page break]*  *(randomize order of image presented – one image of fruit drink with high added sugar & one image of similar fruit drink without added sugar; randomize order of the three questions in this section; see product images in appendix)* |  |
| Unhealthy fruit drink (unhealthier_sug) | In your opinion, which one of these products is MOST unhealthy?  ¿Cuál de estos productos es el MÁS dañino? | 1 = (image of control product)  2 = (image of experimental product)  *(Randomize their order)* |
| High sugar fruit drink (higher_sug) | Which of these products is higher in sugar?  ¿Cuál de estos productos es MÁS alto en azúcar? | 1 = (image of control product)  2 = (image of experimental product)  *(Randomize their order)* |
| Purchase preference (buywhich_sug) | Which of these products would you rather buy?  ¿Cuál de estos productos preferiría comprar?  *[page break]* | 1 = (image of control product)  2 = (image of experimental product)  *(Randomize their order)* |
| **Single Product Assessment Task** | | |
| Prompt [3] | *[one-time prompt]*  **The next questions are about food products. You will look at a few different products and answer questions about each one.**  **Please keep in mind that this study seeks to evaluate your survey responses and not the sale of the product.**  **Las siguientes preguntas son sobre los productos comestibles. Observará algunos productos y responderá preguntas sobre cada uno.**  **Por favor recuerde que este estudio busca evaluar la comprensión del cuestionario y no la venta del producto.**  *(Randomize order of product presented – cookies, yogurt, bread - within each arm)*  *[Repeated prompt with each new messages’ set of questions]*  *[Insert page break]* |  |
| **Cookies** | | |
| Excess saturated fat (excs_sf) | Do you think this product has excess saturated fat?  ¿Cree que este producto tiene exceso de grasa saturada?  *[display image of cookies with label according to study arm]*  *[page break]* | 1 = Yes  0= No  1 = Sí  0 = No  *(display answers in random order)* |
| Prompt | **Stamp refers to the enlarged image coming from upper right corner of the product packaging.**  **El sello se refiere a la imagen aumentada que aparece en la esquina superior derecha del empaque del producto.**  *[display image of cookies with label according to study arm]* |  |
| PME concern saturated fat (pme_conc_sf) [3, 4] | Looking at the stamp, how concerned would you be about the health consequences of consuming this product?  Al ver el sello, ¿Qué tanto se preocuparía usted de las consecuencias en salud al consumir este producto?  *[do not ask this question to the control group]* | 5 = Very much  4 = A lot  3 = A little  2 = Very little  1 = Not at all  5= Muchísimo  4 = Mucho  3 = Poco  2 = Muy poco  1 = Nada  *Note: all items assessed on this scale were originally measured as 1 = Very much … 5 = Not at all and recoded for analysis. These items are coded as per the original scale in the shared data files.* |
| PME unpleasant saturated fat (pme_unpl_sf) [3, 4] | How much does the stamp make consuming this product seem unpleasant to you?  ¿Qué tanto hace el sello que consumir este producto le parezca desagradable?  *[do not ask this question to the control group]* | 5 = Very much  4 = A lot  3 = A little  2 = Very little  1 = Not at all  5= Muchísimo  4 = Mucho  3 = Poco  2 = Muy poco  1 = Nada |
| PME discourage saturated fat (pme_disc_sf) [3, 4] | How much does the stamp discourage you from wanting to consume this product?  ¿Qué tanto lo desanima el sello a consumir este producto?  *[do not ask this question to the control group]*  *[page break]* | 5 = Very much  4 = A lot  3 = A little  2 = Very little  1 = Not at all  5= Muchísimo  4 = Mucho  3 = Poco  2 = Muy poco  1 = Nada |
| Purchase likelihood saturated fat (buy_lkly_sf) | How likely is it for you to want to purchase this product next week, if it were available?  ¿Qué tanto le gustaría comprar este producto la próxima semana, si estuviera disponible? | 5 = Very much  4 = A lot  3 = A little  2 = Very little  1 = Not at all  5= Muchísimo  4 = Mucho  3 = Poco  2 = Muy poco  1 = Nada |
| **Yogurt** | | |
| Excess sugar (excs_sug) | Do you think this product has excess sugar?  ¿Cree que este producto tiene exceso de azúcar?  *[display image of yogurt with label according to study arm]*  *[page break]* | 1 = Yes  0 = No  1 = Sí  0 = No  *(display answers in random order)* |
| Prompt | **Stamp refers to the enlarged image coming from upper right corner of the product packaging.**  **El sello se refiere a la imagen aumentada que aparece en la esquina superior derecha del empaque del producto.**  *[display image of yogurt with label according to study arm]* |  |
| PME concern sugar (pme_conc_sug) [3, 4] | Looking at the stamp, how concerned would you be about the health consequences of consuming this product?  Al ver el sello, ¿Qué tanto se preocuparía usted de las consecuencias en salud al consumir este producto?  *[do not ask this question to the control group]* | 5 = Very much  4 = A lot  3 = A little  2 = Very little  1 = Not at all  5= Muchísimo  4 = Mucho  3 = Poco  2 = Muy poco  1 = Nada |
| PME unpleasant sugar (pme_unpl_ sug) [3, 4] | How much does the stamp make consuming this product seem unpleasant to you?  ¿Qué tanto hace el sello que consumir este producto le parezca desagradable?  *[do not ask this question to the control group]* | 5 = Very much  4 = A lot  3 = A little  2 = Very little  1 = Not at all  5= Muchísimo  4 = Mucho  3 = Poco  2 = Muy poco  1 = Nada |
| PME discourage sugar (pme_disc_ sug) [3, 4] | How much does the stamp discourage you from wanting to consume this product?  ¿Qué tanto lo desanima el sello a consumir este producto?  *[do not ask this question to the control group]*  *[page break]* | 5 = Very much  4 = A lot  3 = A little  2 = Very little  1 = Not at all  5= Muchísimo  4 = Mucho  3 = Poco  2 = Muy poco  1 = Nada |
| Purchase likelihood sugar (buy_lkly_ sug) | How likely is it for you to want to purchase this product next week, if it were available?  ¿Qué tanto le gustaría comprar este producto la próxima semana, si estuviera disponible? | 5 = Very much  4 = A lot  3 = A little  2 = Very little  1 = Not at all  5= Muchísimo  4 = Mucho  3 = Poco  2 = Muy poco  1 = Nada |
| **Bread** | | |
| Excess sodium (excs_sod) | Do you think this product has excess salt/sodium?  ¿Cree que este producto tiene exceso de sal/sodio?  *[display image of bread with label according to study arm]*  *[page break]* | 1 = Yes  0 = No  1 = Sí  0= No  *(display answers in random order)* |
| Prompt | **Stamp refers to the enlarged image coming from upper right corner of the product packaging.**  **El sello se refiere a la imagen aumentada que aparece en la esquina superior derecha del empaque del producto.**  *[display image of bread with label according to study arm]* |  |
| PME concern sodium (pme_conc_sod) [3, 4] | Looking at the stamp, how concerned would you be about the health consequences of consuming this product?  Al ver el sello, ¿Qué tanto se preocuparía usted de las consecuencias en salud al consumir este producto?  *[do not ask this question to the control group]* | 5 = Very much  4 = A lot  3 = A little  2 = Very little  1 = Not at all  5= Muchísimo  4 = Mucho  3 = Poco  2 = Muy poco  1 = Nada |
| PME unpleasant sodium (pme_unpl_ sod) [3, 4] | How much does the stamp make consuming this product seem unpleasant to you?  ¿Qué tanto hace el sello que consumir este producto le parezca desagradable?  *[do not ask this question to the control group]* | 5 = Very much  4 = A lot  3 = A little  2 = Very little  1 = Not at all  5= Muchísimo  4 = Mucho  3 = Poco  2 = Muy poco  1 = Nada |
| PME discourage sodium (pme_disc_ sod) [3, 4] | How much does the stamp discourage you from wanting to consume this product?  ¿Qué tanto lo desanima el sello a consumir este producto?  *[do not ask this question to the control group]*  *[page break]* | 5 = Very much  4 = A lot  3 = A little  2 = Very little  1 = Not at all  5= Muchísimo  4 = Mucho  3 = Poco  2 = Muy poco  1 = Nada |
| Purchase likelihood sodium (buy_lkly_ sod) | How likely is it for you to want to purchase this product next week, if it were available?  ¿Qué tanto le gustaría comprar este producto la próxima semana, si estuviera disponible? | 5 = Very much  4 = A lot  3 = A little  2 = Very little  1 = Not at all  5= Muchísimo  4 = Mucho  3 = Poco  2 = Muy poco  1 = Nada |
| **Cereal** | | |
| Excess sugar cereal (nutr_xsug_cer) | Do you think this product contains excess sugar?  ¿Cree que este producto tiene exceso de azúcar?  *[display image of cereal with label(s) according to study arm]* | 1 = Yes  0 = No  1 = Sí  0 = No  *(display answers in random order)* |
| Excess sodium cereal (nutr_xsod_cer) | Do you think this product contains excess salt/sodium?  ¿Cree que este producto tiene exceso de sal/sodio?  *[page break]* | 1 = Yes  0= No  1 = Sí  0 = No  *(display answers in random order)* |
|  | **“The stamps” refers to the enlarged images coming from upper right corner of the product packaging.**  **Los sellos se refieren a las imágenes aumentadas que aparecen en la esquina superior derecha del empaque del producto.**  *[display image of cereal with label(s) according to study arm]* |  |
| PME concern cereal (pme_conc_cer) [3, 4] | Looking at the stamps, how concerned would you be about the health consequences of consuming this product?  Al ver los sellos, ¿Qué tanto se preocuparía usted de las consecuencias en salud al consumir este producto?  *[do not ask this question to the control group]* | 5 = Very much  4 = A lot  3 = A little  2 = Very little  1 = Not at all  5= Muchísimo  4 = Mucho  3 = Poco  2 = Muy poco  1 = Nada |
| PME unpleasant cereal (pme_unpl_ cer) [3, 4] | How much do these stamps make consuming this product seem unpleasant to you?  ¿Qué tanto hacen los sellos que consumir este producto le parezca desagradable?  *[do not ask this question to the control group]* | 5 = Very much  4 = A lot  3 = A little  2 = Very little  1 = Not at all  5= Muchísimo  4 = Mucho  3 = Poco  2 = Muy poco  1 = Nada |
| PME discourage cereal (pme_disc_cer) [3, 4] | How much do the stamps discourage you from wanting to consume this product?  ¿Qué tanto lo desaniman los sellos a consumir este producto?  *[do not ask this question to the control group]*  *[page break]* | 5 = Very much  4 = A lot  3 = A little  2 = Very little  1 = Not at all  5= Muchísimo  4 = Mucho  3 = Poco  2 = Muy poco  1 = Nada |
| Purchase likelihood cereal (buy_lkly_cer) | How likely is it for you to want to purchase this product next week, if it were available?  ¿Qué tanto le gustaría comprar este producto la próxima semana, si estuviera disponible? | 5 = Very much  4 = A lot  3 = A little  2 = Very little  1 = Not at all  5= Muchísimo  4 = Mucho  3 = Poco  2 = Muy poco  1 = Nada |
| **Label Comparison** | | |
| Prompt | **The next questions are about different labels.**  **Las siguientes preguntas son sobre diferentes sellos.**  *[Randomize the participant to see the items for one of the three nutrients, not all three]*  *[page break]* |  |
| Most discouraging label (most_disc) | Which of these labels would discourage you most from wanting to consume this product?  ¿Cuál de estos sellos lo/la desmotiva MÁS de querer consumir el producto?  *[display image of excess nutrient product w/o label]*  *[page break]* | [answer choices are each of the label types randomly displayed] |
| **Demographics** | | |
| Department (dept) | Which department do you live in? If you live in Bogota, select Bogota.  ¿En qué departamento vive? Si es en Bogotá seleccione Bogotá | [list out 32 departments] – drop down  1=Bogotá  2=Amazonas  3=Antioquia  4=Arauca  5=Atlántico  6=Bolivar  7-Boyacá  8=Caldas  9=Caquetá  10=Casanare  11=Cauca  12=Cesar  13=Chocó  14=Córdoba  15=Cundinamarca  16=Guainía  17=Guaviare  18=Huila  19=La Guajira  20=Magdalena  21=Meta  22=Nariño  23=Norte de Santander  24=Putumayo  25=Quindío  26=Risaralda  27=San Andrés y Providencia  28=Santander  29=Sucre  30=Tolima  31=Valle del Cauca  32=Vaupés  33=Vichada |
| Children (children) | Are you the parent or caregiver of any children (ages 0-18) who currently live in your household?  ¿Es usted padre, madre o cuidador/a de algún niño, niña o adolescente (de 0 a 18 años) que actualmente viva en su hogar?  *[page break]* | 1=Yes  0=No  1=Sí  0=No |
| Number of children (children_hhld) | How many children (ages 0-18) who currently live in your household are you the parent of caregiver of?  ¿De cuántos niños, niñas o adolescentes (de 0 a 18 años) que vivan actualmente en su hogar es usted el cuidador? | ____ [restricted to 1-15]  *[show question if children=1]* |
| Ethnicity (etnia) [5] | According to your culture or ethnicity how do you recognize yourself? (Check all that apply)  ¿De acuerdo con su cultura, pueblo o rasgos físicos, como se reconoce usted? (Marque todo lo que corresponda) | 1 = Indígenous  2 = African descendent  3 = Caucasian  4 = Mestizo  5 = Other  6 = None  1 = Indígena  2 = Afrodescendiente  3 = Blanco  4 = Mestizo  5 = Otro  6 = Ninguno |
| Height (height) | How tall are you? Enter your height in **meters**.  ¿Cuánto mide? Ingrese su altura en **metros**. | [Numerical free response. Allow entries > 1.0m and <2.3m. Allow 2 decimal places.]. |
| Weight (weight) | How much do you weigh? Enter your weight in **kilograms**.  ¿Cuánto pesa? Ingrese su peso en **kilogramos**. | [Numerical free response. Allow entries > 18kg and <408kg]. |
| Financial situation (finance_sit) | According to your current household’s financial situation you would say that:  Respecto a la situación económica de su hogar en este momento, usted diría que:  *[page break]* | 1 = I pay the bills, I can buy necessary things and additional things  2 = I pay the bills and afford to buy what I need  3 = To pay the bills, I have to stop buying things I need  4 = I can’t pay the bills  1 = Pago las facturas, puedo comprar cosas necesarias y otras adicionales.  2 = Pago las facturas y compro apenas lo necesario.  3 = Para pagar las facturas tengo que dejar de comprar cosas que necesito.  4 = No puedo pagar las facturas. |

**References**

1. Ministerio de Educación Nacional. Sistema educativo colombiano: Gobierno de Colombia; 2022 [updated March 15, 2022; cited 2023 January 9]. Available from: <https://www.mineducacion.gov.co/1780/w3-article-233839.html?_noredirect=1>.

2. Ministerio de Educación Nacional. Revisión de políticas nacionales de educación: La educación en Colombia: Gobierno de Colombia; 2016 [cited 2023 January 9]. Available from: <https://www.mineducacion.gov.co/1759/articles-356787_recurso_1.pdf>.

3. Grummon AH, Hall MG, Taillie LS, Brewer NT. How should sugar-sweetened beverage health warnings be designed? A randomized experiment. Prev Med. 2019;121:158-66. Epub 2019/02/18. doi: 10.1016/j.ypmed.2019.02.010. PubMed PMID: 30772370; PubMed Central PMCID: PMCPMC6520104.

4. Baig SA, Noar SM, Gottfredson NC, Boynton MH, Ribisl KM, Brewer NT. UNC Perceived Message Effectiveness: Validation of a Brief Scale. Ann Behav Med. 2019;53(8):732-42. Epub 2018/10/16. doi: 10.1093/abm/kay080. PubMed PMID: 30321252; PubMed Central PMCID: PMCPMC6636889.

5. Departamento administrativo nacional de estadística. Censo nacional de población y vivienda 2018 Bogotá D.C., Colombia: Gobierno de Colombia; 2022 [cited 2023 January 9]. Available from: <https://www.dane.gov.co/index.php/estadisticas-por-tema/demografia-y-poblacion/censo-nacional-de-poblacion-y-vivenda-2018>.

**Appendix.** Study stimuli.


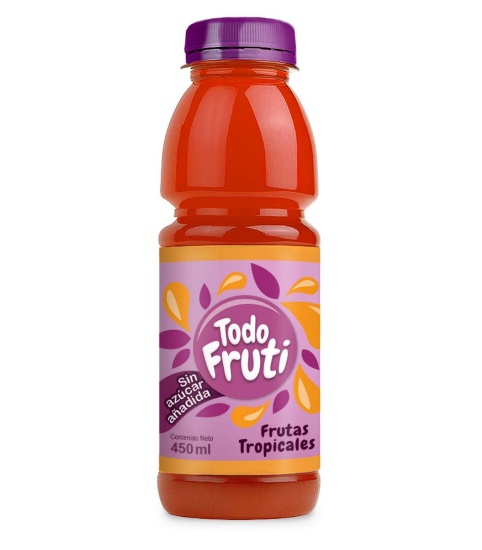

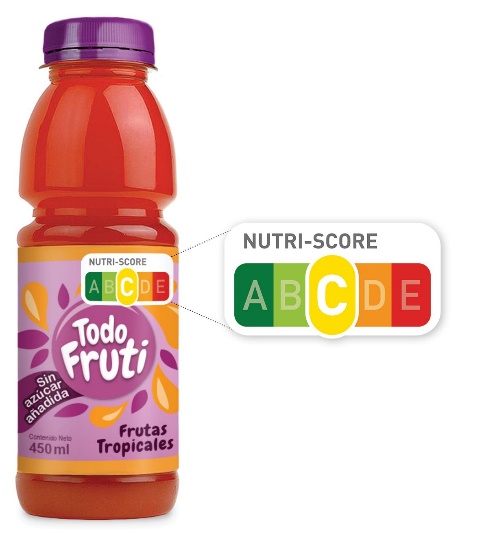

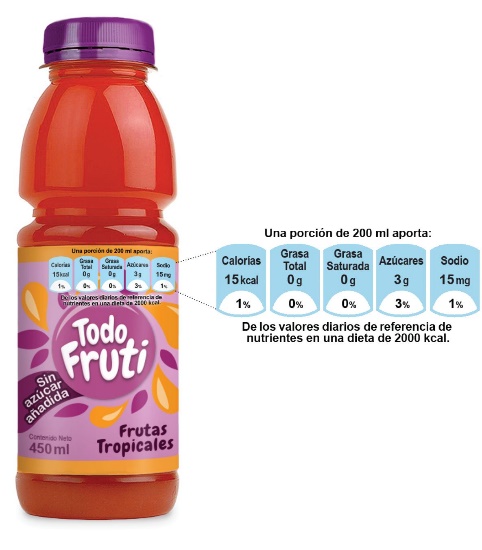


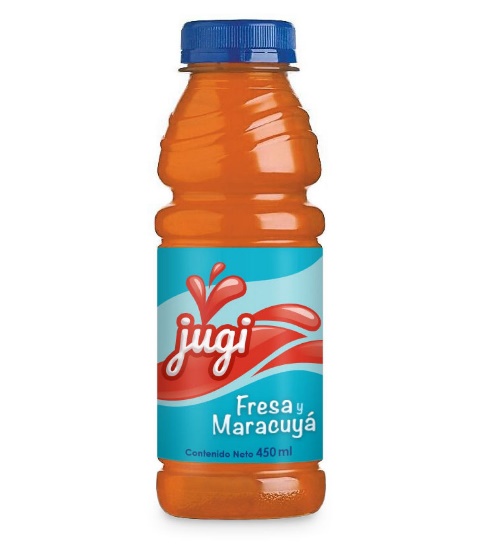

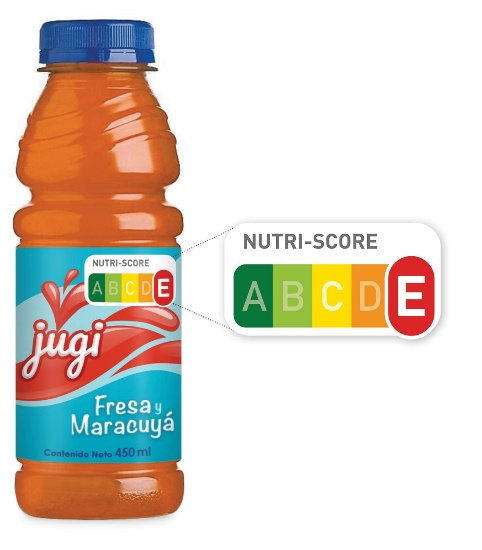

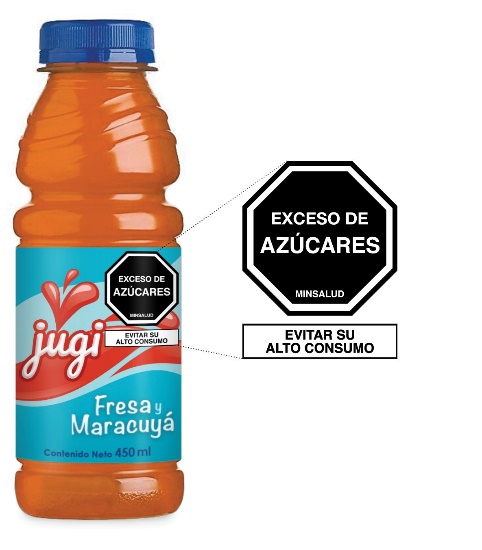

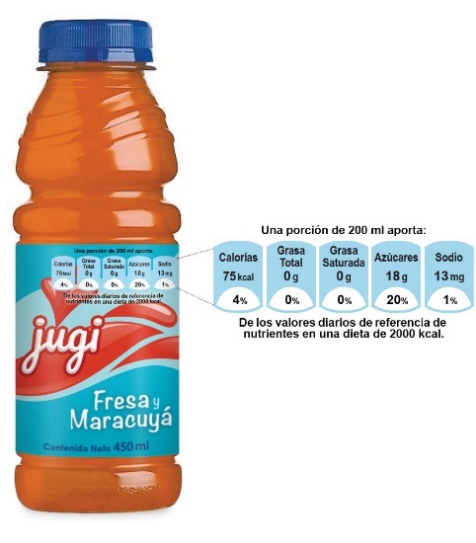


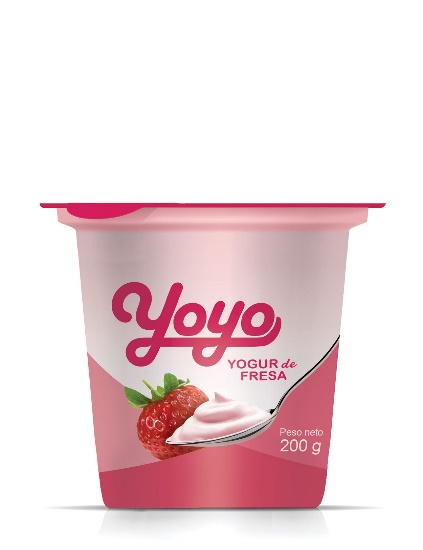

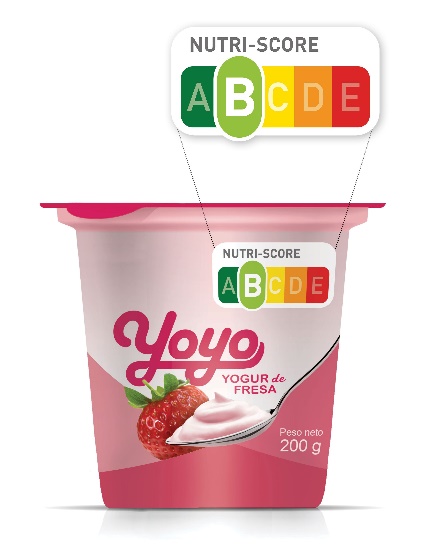

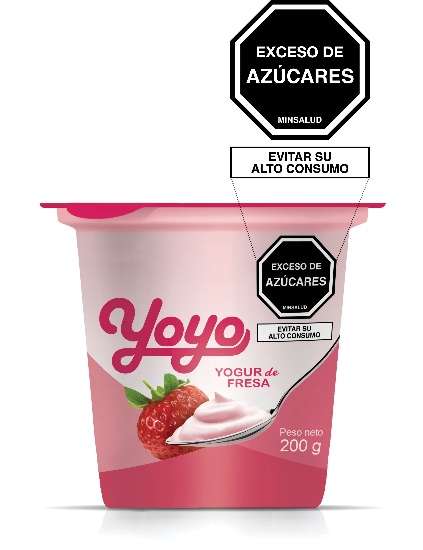

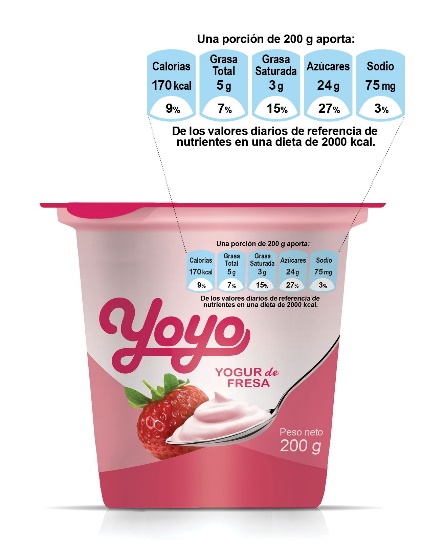


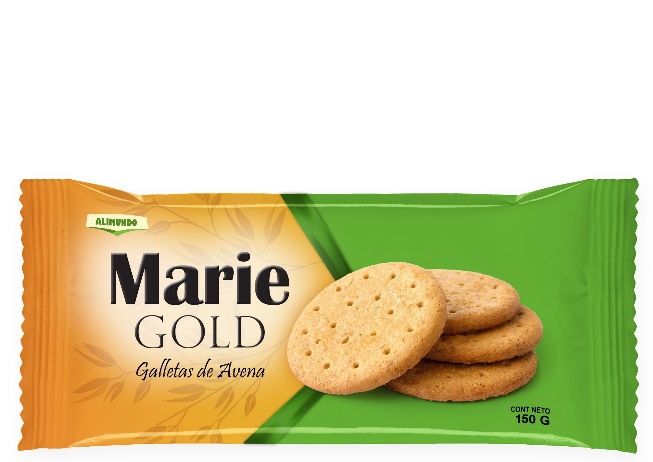

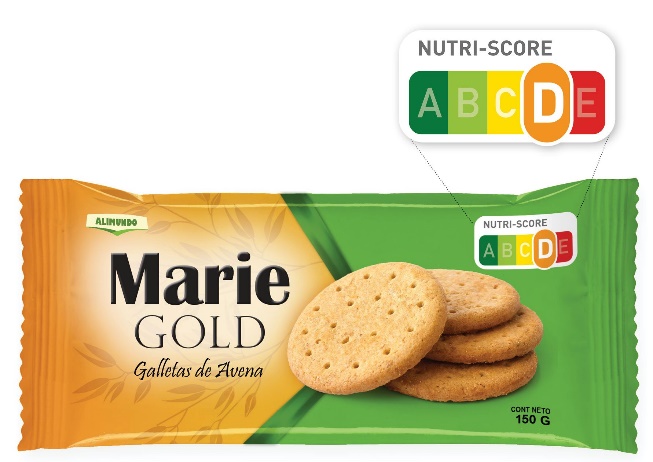

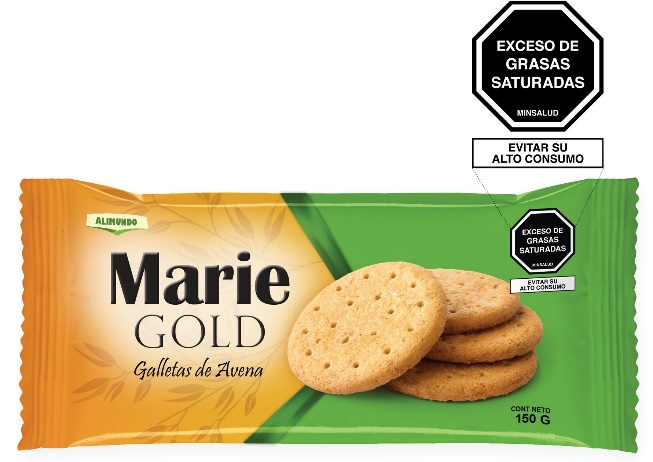


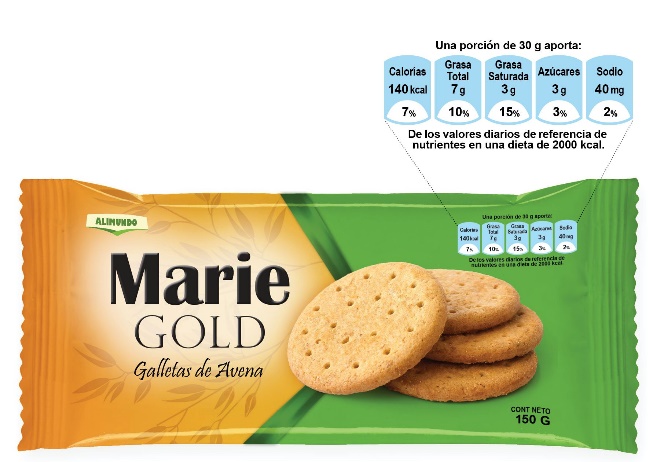


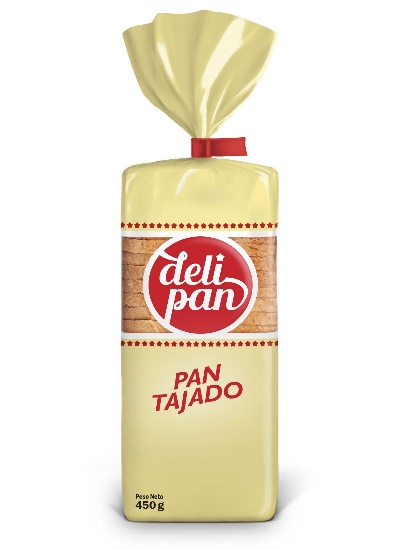

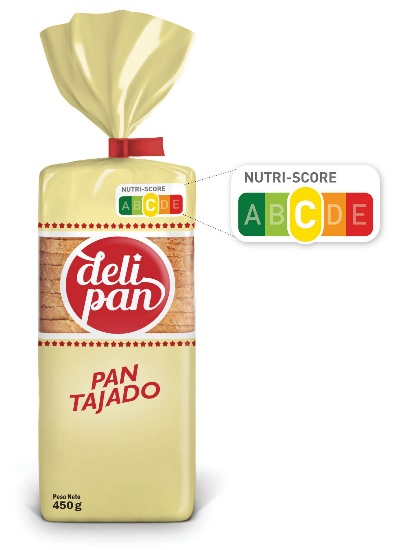

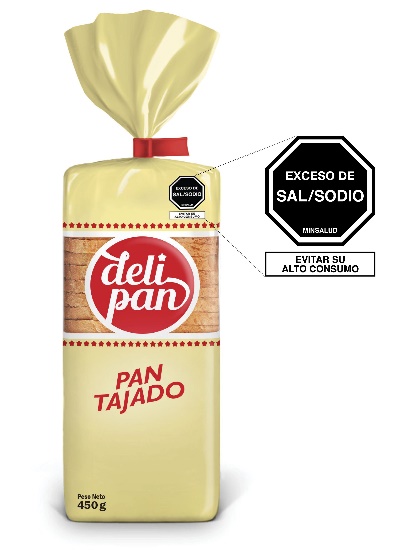

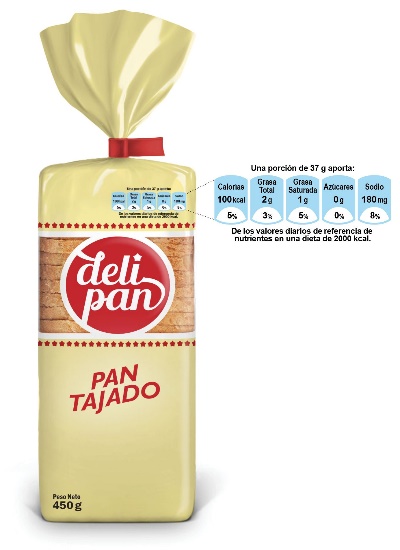


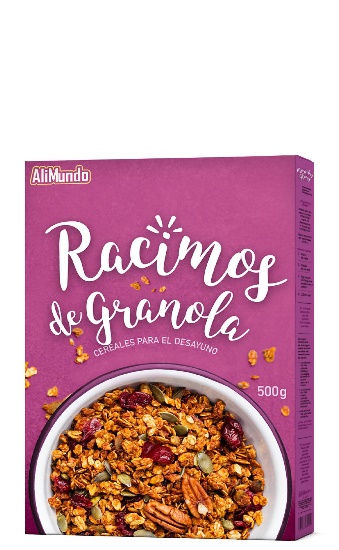

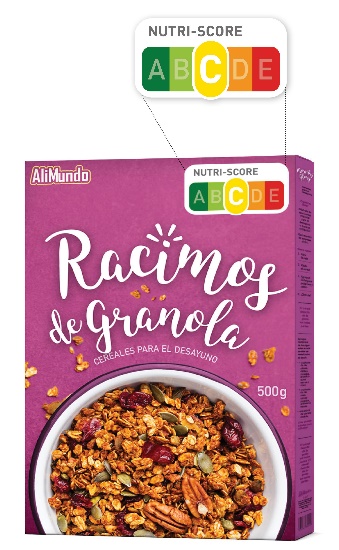

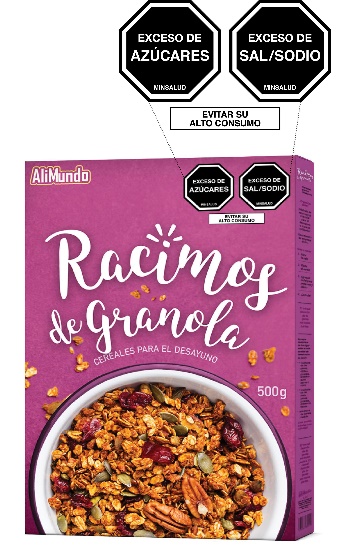

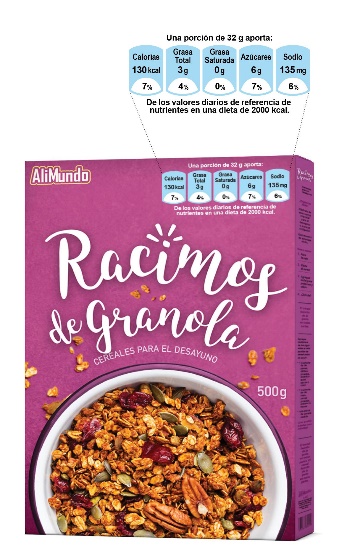

Supplement: S2 Table — (DOCX) [file pone.0303514.s003.docx]
