# Supplementary material for: Impact of nutrient warning labels on Colombian consumers’ selection and identification of food and drinks high in sugar, sodium, and saturated fat: A randomized controlled trial
Source: PLoS One. 2024 Jun 10;19(6):e0303514. doi: 10.1371/journal.pone.0303514 (PMC11164358; doi:10.1371/journal.pone.0303514)
Supplement: S3 Table — *P-values (except for the joint significance of the interaction terms) are for the difference in the contrast with nutrient warning (the reference) by education level (e.g., for no label, the difference between the contrast for secondary or lower, 30.4–16.2, and the contrast for tertiary, 31.3–17). Thus, the effects of no label, Nutri-score, and GDA relative to nutrient warning did not significantly differ by education level. Data analyzed by logistic regressions of the outcomes on indicator variables for the arm, education level, and their interactions. Inference on the contrasts is based on the delta method. Missing data were as follows: 40 (0.5%) for ‘Wanted to purchase the less healthy drink’ (11 in nutrient warning, 12 in no label, 9 in Nutri-score, and 8 in GDA) and 16 (0.2%) for ‘Correctly identified the less healthy fruit drink as higher in sugar’ (4 in nutrient warning, 3 in No label, 8 in Nutri-score, and 1 in GDA). (DOCX) [file pone.0303514.s004.docx]

|  | **Wanted to purchase the less healthy fruit drink**  **(n=7,964)** | | **Correctly identified the less healthy fruit drink as higher in sugar**  **(n=7,988)** | |
| --- | --- | --- | --- | --- |
|  | **%** | **p*** | **%** | **p*** |
| **Nutrient warning** |  |  |  |  |
| Secondary or lower | 16.2 | Ref. | 89.6 | Ref. |
| Tertiary | 17.0 |  | 87.9 |  |
| **No label** |  |  |  |  |
| Secondary or lower | 30.4 | 0.979 | 69.0 | 0.739 |
| Tertiary | 31.3 |  | 68.1 |  |
| **Nutri-Score** |  |  |  |  |
| Secondary or lower | 29.5 | 0.077 | 65.6 | 0.074 |
| Tertiary | 25.5 |  | 68.5 |  |
| **GDA** |  |  |  |  |
| Secondary or lower | 16.2 | 0.074 | 91.9 | 0.158 |
| Tertiary | 12.8 |  | 92.9 |  |
| **Arm × Education (Wald test of joint significance)** |  | 0.091 |  | 0.267 |

*P-values (except for the joint significance of the interaction terms) are for the difference in the contrast with nutrient warning (the reference) by education level (e.g., for no label, the difference between the contrast for secondary or lower, 30.4 – 16.2, and the contrast for tertiary, 31.3 – 17). Thus, the effects of no label, Nutri-Score, and GDA relative to nutrient warning did not significantly differ by education level. Data analyzed by logistic regressions of the outcomes on indicator variables for the arm, education level, and their interactions. Inference on the contrasts is based on the delta method. Missing data were as follows: 40 (0.5%) for ‘Wanted to purchase the less healthy drink’ (11 in nutrient warning, 12 in no label, 9 in Nutri-Score, and 8 in GDA) and 16 (0.2%) for ‘Correctly identified the less healthy fruit drink as higher in sugar’ (4 in nutrient warning, 3 in No label, 8 in Nutri-Score, and 1 in GDA).
