# Supplementary material for: Impact of nutrient warning labels on Colombian consumers’ selection and identification of food and drinks high in sugar, sodium, and saturated fat: A randomized controlled trial
Source: PLoS One. 2024 Jun 10;19(6):e0303514. doi: 10.1371/journal.pone.0303514 (PMC11164358; doi:10.1371/journal.pone.0303514)
Supplement: S4 Table — Standard errors in parentheses. Data analyzed at the participant level. (DOCX) [file pone.0303514.s005.docx]

|  | Wanted to purchase the less healthy fruit drink | | Correctly identified the less healthy fruit drink as higher in sugar | | Correctly identified the less healthy fruit drink as less healthy |
| --- | --- | --- | --- | --- | --- |
|  | (1) | (2) | (1) | (2) | (1) |
| No label | 0.805 | 0.816 | -1.280 | -1.356 | -1.341 |
|  | (0.076) | (0.115) | (0.085) | (0.130) | (0.086) |
| Nutri-Score | 0.628 | 0.771 | -1.338 | -1.512 | -1.161 |
|  | (0.080) | (0.120) | (0.086) | (0.134) | (0.088) |
| Warning label | (ref) | (ref) | (ref) | (ref) | (ref) |
|  |  |  |  |  |  |
| GDA | -0.180 | 0.003 | 0.449 | 0.276 | -0.152 |
|  | (0.089) | (0.132) | (0.112) | (0.169) | (0.100) |
| Up to secondary |  | (ref) |  | (ref) |  |
|  |  |  |  |  |  |
| Tertiary |  | 0.059 |  | -0.170 |  |
|  |  | (0.123) |  | (0.145) |  |
| No label # Tertiary |  | -0.018 |  | 0.130 |  |
|  |  | (0.154) |  | (0.172) |  |
| Nutri-Score # Tertiary |  | -0.256 |  | 0.304 |  |
|  |  | (0.160) |  | (0.175) |  |
| GDA # Tertiary |  | -0.333 |  | 0.307 |  |
|  |  | (0.179) |  | (0.226) |  |
| Intercept | -1.611 | -1.645 | 2.058 | 2.156 | 2.094 |
|  | (0.061) | (0.092) | (0.071) | (0.111) | (0.072) |
| N | 7,964 | 7,964 | 7,988 | 7,988 | 7,978 |

Standard errors in parentheses. Data analyzed at the participant level. Missing data were as follows: 40 (0.5%) for ‘Wanted to purchase the less healthy fruit drink’ (11 in nutrient warning, 12 in no label, 9 in Nutri-Score, and 8 in GDA), 16 (0.2%) for ‘Correctly identified the less healthy fruit drink as higher in sugar’ (4 in nutrient warning, 3 in no label, 8 in Nutri-Score, and 1 in GDA), and 26 (0.3%) for ‘Correctly identified the less healthy fruit drink as less healthy’ (4 in nutrient warning, 15 in no label, 4 in Nutri-Score, and 3 in GDA).
