# Supplementary material for: Impact of nutrient warning labels on Colombian consumers’ selection and identification of food and drinks high in sugar, sodium, and saturated fat: A randomized controlled trial
Source: PLoS One. 2024 Jun 10;19(6):e0303514. doi: 10.1371/journal.pone.0303514 (PMC11164358; doi:10.1371/journal.pone.0303514)
Supplement: S5 Table — (DOCX) [file pone.0303514.s006.docx]

|  | **No label** | | **Nutri-Score** | | **Nutrient warning** | | **GDA** | |
| --- | --- | --- | --- | --- | --- | --- | --- | --- |
| **Correctly identified all excess nutrients, n (% of non-missing)** |  |  |  |  |  |  |  |  |
| Cookies | 557 | (25) | 847 | (44) | 1,494 | (76) | 809 | (42) |
| Yogurt | 1,191 | (54) | 829 | (43) | 1,722 | (88) | 1,541 | (80) |
| Bread | 810 | (37) | 772 | (40) | 1,598 | (82) | 1,168 | (61) |
| Cereal | 287 | (13) | 383 | (20) | 1,357 | (69) | 328 | (17) |
| Total | 2,845 | (32) | 2,831 | (37) | 6,171 | (79) | 3,846 | (50) |
| **Likelihood of purchasing the product next week if available, mean (SD)** |  |  |  |  |  |  |  |  |
| Cookies | 3.55 | (0.95) | 3.29 | (1.00) | 2.77 | (1.11) | 3.35 | (0.96) |
| Yogurt | 3.50 | (0.96) | 3.57 | (0.95) | 2.79 | (1.11) | 3.16 | (1.03) |
| Bread | 3.34 | (0.94) | 3.34 | (0.95) | 2.74 | (1.09) | 3.22 | (0.97) |
| Cereal | 3.72 | (0.99) | 3.45 | (1.03) | 2.77 | (1.18) | 3.44 | (1.01) |
| Total | 3.53 | (0.97) | 3.41 | (0.99) | 2.77 | (1.12) | 3.29 | (1.00) |
| **PME, mean (SD)** |  |  |  |  |  |  |  |  |
| Cookies |  |  | 3.02 | (1.01) | 3.70 | (0.97) | 3.08 | (0.95) |
| Yogurt |  |  | 2.74 | (0.96) | 3.74 | (0.97) | 3.37 | (0.96) |
| Bread |  |  | 2.94 | (0.93) | 3.73 | (0.94) | 3.20 | (0.97) |
| Cereal |  |  | 2.88 | (0.97) | 3.81 | (0.97) | 3.08 | (0.99) |
| Total |  |  | 2.89 | (0.97) | 3.75 | (0.96) | 3.18 | (0.98) |

Missing data at the participant-product level were as follows: 47 (0.1%) for ‘Correctly identified product as having excess nutrients’ (16 in nutrient warning, 5 in no label, 12 in Nutri-Score, and 14 in GDA), 142 (0.4%) for ‘Likelihood to purchase the product in the next week if it were available’ (36 in nutrient warning, 23 in no label, 37 in Nutri-Score, and 46 in GDA), and 116 (0.5%) for ‘Perceived message effectiveness (PME)’ (36 in nutrient warning, 38 in Nutri-Score, and 42 in GDA).
