# Supplementary material for: Impact of nutrient warning labels on Colombian consumers’ selection and identification of food and drinks high in sugar, sodium, and saturated fat: A randomized controlled trial
Source: PLoS One. 2024 Jun 10;19(6):e0303514. doi: 10.1371/journal.pone.0303514 (PMC11164358; doi:10.1371/journal.pone.0303514)
Supplement: S6 Table — Standard errors in parentheses. PME not measured in No label. No residual variance for ‘Correctly identified product as having excess nutrients’ (multilevel mixed-effects logistic regression). Data analyzed at the participant-product level. (DOCX) [file pone.0303514.s007.docx]

|  | Perceived message effectiveness (PME) | Correctly identified product as having excess nutrients | Likelihood of purchasing the product in the next week if it were available |
| --- | --- | --- | --- |
| No label | n/a | -3.103 | 0.780 |
|  |  | (0.100) | (0.032) |
| Nutri-Score | -0.683 | -1.960 | 0.521 |
|  | (0.031) | (0.097) | (0.033) |
| Warning label | (ref) | (ref) | (ref) |
|  |  |  |  |
| GDA | -0.624 | -2.069 | 0.577 |
|  | (0.031) | (0.097) | (0.033) |
| Cookies | (ref) | (ref) | (ref) |
|  |  |  |  |
| Yogurt | 0.044 | 1.074 | 0.023 |
|  | (0.019) | (0.101) | (0.024) |
| Bread | 0.032 | 0.435 | -0.026 |
|  | (0.019) | (0.092) | (0.024) |
| Cereals | 0.110 | -0.510 | 0.006 |
|  | (0.019) | (0.086) | (0.024) |
| No label # Yogurt | n/a | 0.638 | -0.067 |
|  |  | (0.127) | (0.032) |
| No label # Bread | n/a | 0.298 | -0.184 |
|  |  | (0.120) | (0.032) |
| No label # Cereals | n/a | -0.546 | 0.168 |
|  |  | (0.125) | (0.032) |
| Nutri-Score # Yogurt | -0.321 | -1.125 | 0.254 |
|  | (0.028) | (0.126) | (0.033) |
| Nutri-Score # Bread | -0.106 | -0.650 | 0.073 |
|  | (0.028) | (0.120) | (0.034) |
| Nutri-Score # Cereals | -0.244 | -1.017 | 0.158 |
|  | (0.028) | (0.120) | (0.034) |
| GDA # Yogurt | 0.250 | 1.224 | -0.205 |
|  | (0.028) | (0.132) | (0.033) |
| GDA # Bread | 0.088 | 0.602 | -0.096 |
|  | (0.028) | (0.120) | (0.034) |
| GDA # Cereals | -0.102 | -1.163 | 0.093 |
|  | (0.028) | (0.123) | (0.034) |
| Intercept | 3.699 | 1.637 | 2.769 |
|  | (0.022) | (0.073) | (0.023) |
| Participant-level error variance | 0.567 | 2.059 | 0.487 |
|  | (0.012) | (0.086) | (0.010) |
| Residual variance | 0.367 | n/a | 0.543 |
|  | (0.004) |  | (0.005) |
| N | 23,132 | 31,969 | 31,874 |

Standard errors in parentheses. PME not measured in No label. No residual variance for ‘Correctly identified product as having excess nutrients’ (multilevel mixed-effects logistic regression). Data analyzed at the participant-product level. Missing data were as follows: 47 (0.1%) for ‘Correctly identified product as having excess nutrients’ (16 in nutrient warning, 5 in no label, 12 in Nutri-Score, and 14 in GDA), 142 (0.4%) for ‘Likelihood to purchase the product in the next week if it were available’ (36 in nutrient warning, 23 in no label, 37 in Nutri-Score, and 46 in GDA), and 116 (0.5%) for ‘Perceived message effectiveness (PME)’ (36 in nutrient warning, 38 in Nutri-Score, and 42 in GDA).
