# Supplementary material for: Impact of nutrient warning labels on Colombian consumers’ selection and identification of food and drinks high in sugar, sodium, and saturated fat: A randomized controlled trial
Source: PLoS One. 2024 Jun 10;19(6):e0303514. doi: 10.1371/journal.pone.0303514 (PMC11164358; doi:10.1371/journal.pone.0303514)
Supplement: Attachment — Submitted filename: PLOS One Response to Reviewers_3.29.24.docx [file pone.0303514.s008.docx]

April 1, 2024

Dear editors and reviewers,

Thank you for your review and comments. We have included specific responses to your comments below. We believe that these revisions have resulted in a stronger presentation of our work. We note a few major changes including:

- We have included further detail on how we reached the final analytic sample of 8,004.
- We have more clearly stated the primary and secondary outcomes of interest in the background section.
- We have added additional information about missing data.

We look forward to your further review.

Best,

Lindsey Smith Taillie on behalf of co-authors

**Ms. Ref. No.:** PONE-D-23-37686

**Title:** “Impact of nutrient warning labels on Colombian consumers’ selection and identification of food and drinks high in sugar, sodium, and saturated fat: A randomized controlled trial”

| **#** | **Reviewers’ comments** | **Revision or Response** | **Line Number** |
| --- | --- | --- | --- |
|  | **Academic Editor** |  |  |
| 1 | Please ensure that your manuscript meets PLOS ONE's style requirements, including those for file naming. | We have reviewed the manuscript and ensured that it meets PLOS ONE’s style requirements. | N/A |
| 2 | Please review your reference list to ensure that it is complete and correct. If you have cited papers that have been retracted, please include the rationale for doing so in the manuscript text, or remove these references and replace them with relevant current references. Any changes to the reference list should be mentioned in the rebuttal letter that accompanies your revised manuscript. If you need to cite a retracted article, indicate the article’s retracted status in the References list and also include a citation and full reference for the retraction notice. | We have reviewed our reference list and confirm that it is complete and correct.  We have one retracted article cited, which is our original article that the current paper is replacing via the replication. We have updated the reference to clearly state the article is retracted. The rationale for including the citation is reported in the text. We state:  *“To inform this discussion, we originally published results from a randomized experiment testing front-of-package labels in Colombia in PLOS ONE in 2022 (doi: 10.1371/journal.pone.0263324) [29], and after publication, the study authors and a reader, independently noticed and then notified PLOS ONE about errors in the calculation of the nutritional information used in the mock front-of-package labels used in this study. We decided to retract our original article [cite retraction once published] and replicated the original study following an identical study protocol and identical sampling procedures, using corrected nutrition labels. This replication study included an additional quality control process in which all nutrition labels were independently created by two dietitians and then compared to ensure all steps of the nutrition label creation process were correct.”*  Of note, the article has not been officially retracted, but it will be when this current manuscript is published. We defer to the journal’s preferences on whether and how to cite the article once retracted. | 111-120; 620 |
| 3 | 1- Better explain the limitations due to use of an online sample. Groups are well balanced but information of how many of the 300,000 panel participants in Colombia were invited should be provided. Also, after randomization about 20% was lost (did not complete the questionaries). Exclusions only apply for those before randomization | The number of panel participants invited to participate is not collected by Offerwise, so we are unable to include this information in the paper. We have also updated the flow chart to clearly report the number of participants who dropped out of the study and those who were excluded for specific reasons. We have added text to the manuscript to provide further detail about the participants who were analyzed but not in the final analytic sample:  *“We randomized 10,160 participants with a final analytical sample size of 8,004 (Fig 2). After randomization, 1,999 participants did not complete the study; we used per protocol analysis, so these participants were not included in our analytic sample. Additionally, 157 participants were excluded from analyses for any of the following reasons: missing all outcome data (n=2), invalid age (n=6), completing the study in less than two minutes (2), and repeat or overlapping responses (n=147). In these cases, we dropped repeat responses (keeping each participant’s first response) if they did not overlap and dropped all responses (including the first) if they did overlap.”* | 306-312 |
| 4 | 2- I am unaware of using repeated measures for comparisons for different products as follows “For outcomes that were assessed using repeated measures for multiple product types, we used mixed models treating the intercept as ……” These models included the between-subjects factor (i.e., label type), the within-subjects factor (i.e., product type), and their interaction. (table 3). | In the second task, participants were shown four products, one at a time, and answered the same set of questions for each product. This led to repeated measures because the survey collected multiple observations per participant for each variable (outcome), with the measures repeated for multiple products rather than over time. Our models add a participant-level component to the error term to account for correlation between each participant’s responses across these products. This error component could be equally construed as accounting for within-participant correlation over the time points characterized by each product display. We estimated single models for the pooled responses rather than separate models for each product to obtain estimates of the average effects of each condition across all four products. | N/A |
| 5 | Supplementary table 4 is more informative than the table 3. Correctly identified product as having excess nutrients. Values are almost the same: 79 32 37 50 vs 78.7, 32.5, 36.9 and 50. I could not understand the meaning of the interaction in this analysis. | Supplementary table 4 includes unadjusted means and proportions by label type for the single product assessment tasks, while table 3 reflects our model-based results. The values are slightly different due to rounding. In supplementary table 4, we rounded to the tenths place, while in table 3 we rounded to the ones place. We have decided to update supplementary table 4 and round to the ones place for percentages like we did in table 3.  Regarding the interaction results reported in supplementary table 3 - the purpose of supplementary table 3 is to describe whether the outcomes “Wanted to purchase the less healthy fruit drink” and “Correctly identified the less healthy fruit drink as higher in sugar” differed by education level (secondary or lower v. tertiary). We have added additional text to footnotes of supplementary table 3 stating:  *“Thus, the effects of no label, Nutri-score, and GDA relative to nutrient warning did not significantly differ by education level.”* | N/A |
|  | **Reviewer #1** |  |  |
| 6 | Lines 129-132, the primary and secondary objectives are to be clearly outlined based on primary and secondary outcomes. | We have modified the background to explicitly state the pre-registered primary and secondary outcomes.  *“Specifically, the primary outcomes were 1) selection of the product high in sugar as the product the participant would rather buy, and 2) correct identification of the product higher in sugar. Secondary outcomes included perceived message effectiveness of the labels, likelihood of purchasing the product in the next week, ability to identify the less healthy product, ability to identify the product with excess of a nutrient of concern, and which label was perceived as most discouraging.”* | 125-130 |
| 7 | Line 305, the outcomes are to be clearly stated. Whether separate calculations for each primary outcome and between each arm were carried out and whether one with the largest sample size was chosen is to be clearly stated. More information is to be provided in this section. | The primary and secondary outcomes are specified in the introduction, lines 125-130:  *“Specifically, the primary outcomes were 1) selection of the product high in sugar as the product the participant would rather buy, and 2) correct identification of the product higher in sugar. Secondary outcomes included perceived message effectiveness of the labels, likelihood of purchasing the product in the next week, ability to identify the less healthy product, ability to identify the product with excess of a nutrient of concern, and which label was perceived as most discouraging.”*  We have provided additional information about how the sample size was selected.  *“A two-sided critical alpha of 0.05 was used to assess statistical significance. Prior to the study, we used G.Power 3.1.9.4 to estimate that with a sample of 8,000 (2,000 per arm), alpha of 0.05, and 80% power, we would be able to detect a small effect of d=0.09 for both primary outcomes (two-sided two-sample t-test for means).”* | 301-304 |
| 8 | A write-up on missing data is to be provided in the method section. | We have added the following at the end of the methods section: *“In the analytic sample, analyses were conducted on a complete-case basis. In the single product assessment task where outcomes were analyzed at the participant-product level, we used all available data from any complete participant-product observation.*  Additionally, we have added information on missing data in the notes to Table 3 and Figures 3 and 4, and we have modified the text on the calculation of the PME variable to clarify that it was calculated only for people with complete data on PME:  *“For the secondary outcome of PME, we re-coded variables so that a higher score represented a higher perceived effectiveness and calculated it at the participant-product level as the average of the three items for each product type (Cronbach's alpha for each product type >.70) if all items were non-missing. PME was not calculated for participant-product observations with any PME item missing.”* | 312-315; 317-321; 355-359; 402-407; 418-419 |
| 9 | Per protocol analysis is to be stated. | We have added that the analysis was per protocol in the text. We have added the following statement where we describe the analytic sample: *“We used per protocol analysis, so these participants were not included in our analytic sample.”* | 307-308 |
| 10 | Line 291, the type/name of random method is to be stated. | We have specified the randomization method in the text.  *“Finally, participants were randomly assigned by Qualtrics using a simple allocation ratio to see the yogurt, cookies, or sliced bread again (one product only).”* | 290-291 |
| 11 | For Table 3 SE (pp), pp is to be omitted. | Thank you for noticing this error. We have removed “pp” from the table header. | N/A |
| 12 | Linear regression, logistic regression, and mixed models which were mentioned in the analyses section in the methodology are to be presented with detailed output and denoted in the tables/figures footnotes in the results section. | We have added information on how data were analyzed in the notes to Table 3 and Figures 3 and 4, and the statistical output in the supplement (Tables S5 and S6). | 353-354; 398-402; 417-418 |
| 13 | S4 Table, n to be inserted apart from %. | Currently in S4 Table we report the n and the percentage apart, such that the format is n (%). | N/A |
| 14 | S3 Table, n to be provided. The nutrient warning as a reference is to be indicated in the table footnote. | We have added the n values to S3 Table, and we have indicated that nutrient warning is the reference group in the table footnote. | N/A |
| 15 | References did not conform to the journal format. | We have reviewed and edited all references and confirm that they conform to the journal format. | N/A |
